# Phosphatase SHP1 impedes mesenchymal stromal cell immunosuppressive capacity modulated by JAK1/STAT3 and P38 signals

**DOI:** 10.1186/s13578-020-00428-w

**Published:** 2020-05-14

**Authors:** Menghui Jiang, Jiayin Ye, Xuefeng Wang, Na Li, Ying Wang, Yufang Shi

**Affiliations:** 1 School of Public Health, Qingdao University, Qingdao, China; 2 The Third Affiliated Hospital of Soochow University, Institutes for Translational Medicine, Soochow University, Suzhou, China; 3 Key Laboratory of Stem Cell Biology, Shanghai Institute of Nutrition and Health, Chinese Academy of Sciences, Shanghai, 200031 China

**Keywords:** MSCs, SHP1, Immunosuppression, Hepatitis

## Abstract

**Background:**

Mesenchymal stromal cells (MSCs) are multiple stromal cells existing in various tissues and have already been employed in animal models and clinical trials to treat immune disorders through potent immunosuppressive capacity. Our previous reports have suggested that MSC immunosuppression is not intrinsic but is acquired upon combined inflammatory cytokine treatment. However, the understanding of detailed molecular mechanisms involved in MSC immunomodulation remains incomplete.

**Results:**

In the study, we report that MSCs derived from *viable motheaten* (*me*^*v*^) mice, with deficiency in SH2 domain-containing phosphatase-1 (SHP1), exhibited remarkable increased suppressive effect on activated splenocyte proliferation. Consistently, when MSCs were treated with combined inflammatory cytokines, SHP1-deficient MSCs produced dramatically more iNOS expression compared with wild-type MSCs. SHP1 was found to suppress the phosphorylation of JAK1/STAT3 and P38 signals. The classical animal model of concanavalin A (ConA)-induced liver injury was applied to examine the role of SHP1 in modulation MSC-therapeutic effect in vivo. Consistent with the results in vitro, SHP1-deficient MSCs exhibited dramatically more effective protection against ConA-induced hepatitis, compared to WT MSCs.

**Conclusion:**

Taken together, our study reveals a possible role for SHP1 in modulation of MSC immunosuppression regulated by JAK1/STAT3 and P38 signals.

## Background

Mesenchymal stromal cells (MSCs) are multipotent fibroblast-like cells which derive from extensive tissue sources. MSCs were first identified in bone marrow derived cells [1], and subsequently were also found in a wide range of other tissues, including adipose tissue [2], umbilical cord [3], dental pulp [4] and so on. MSCs recently have attracted considerable attention for potential in treatment of various immune disorders and degenerative diseases in both animals models and humans, due to their immunoregulatory capacity and differentiation ability [5–7]. MSCs have been shown to effectively suppress T cell and B cell proliferation, monocyte/macrophage polarization, dendritic cell maturation and NK cell cytotoxicity [8–10]. The effect of MSCs on immune cells has been shown to be crucial in mediating MSC-based therapy [11].

MSCs were not intrinsic immunosuppressive, but acquire the capability after activated with IFNγ combined with TNFα, IL1α or IL1β [12]. Recently, MSCs were found to be closely related with immune system in the microenvironment through chemokines and various effective soluble molecules, such as nitric oxide (NO), prostaglandin E2 (PGE2), indoleamine 2,3-dioxygenase (IDO), IL10, transforming growth factor-β1 (TGFβ1), Tumor necrosis factor a (TNFα)-stimulated gene 6 protein (TSG6), Programmed death-ligand 1 (PD-L1) and so on [13–16]. Our further study revealed that mouse and human MSCs perform their immunomodulation mainly through iNOS and IDO respectively [15, 17]. NO produced by iNOS is a major mediator in T cell suppression by mouse MSCs [12]. Secretion of IDO by human MSCs is able to inhibit growth and function of immune cells, by conversing tryptophan to kynurenine [18].

Because of the powerful immunosuppressive capacity, MSCs have already been permitted to be used in clinical trials. Unfortunately, MSCs usually were unable effectively attenuate the symptoms, when came to phase III clinical trials, on account of insufficient immunosuppressive capability of transplanted cells [19]. A better understand of the underlying mechanisms of MSC-based therapeutic activity and the modulation of immunosuppressive capability is highly desired through conducting further extensive clinical and basic studies. In addition, MSCs mostly were applied in chronic diseases, such as systemic lupus erythematosus (SLE) [20], multiple sclerosis (MS) [21], whereas the therapeutic effect of MSCs on acute inflammation is little determined.

SH2-containing protein tyrosine phosphatase 1 (SHP1), as an intracellular protein tyrosine phosphate, contains two tandem SH2 domain at its amino terminus followed by a catalytic domain. SHP1 is predominantly expressed in hematopoietic cells and traditionally considered to negatively regulate hematopoietic and immune cell function through suppress a variety of cytokine signaling systems [22]. According to our previous study, loss of SHP1 in MSCs favored the adipogenic differentiation [23]. In macrophages, SHP1 has been observed to inhibit iNOS expression [24]. Various intrinsic signaling pathways that target iNOS promoter have been suggested to regulate iNOS expression, the most important being Janus kinase/signal transducers and activators of transcription (JAK/STAT) and mitogen-activated protein kinases (MAPKs) signaling. JAK/STAT pathway is essential for iNOS expression, since macrophages derived from STAT1-deficient mice fail to express iNOS and to produce NO [25]. Moreover, inhibition of P38 activation in mouse astrocytes results in a blockade of iNOS expression [26].

Therefore, given the importance of SHP1, in the current study we investigated the function of SHP1 in MSC immunosuppression. We found that SHP1 limited immunosuppression capacity of MSCs through regulating iNOS expression. The mechanism by which SHP1 inhibits these cytokine pathways is being specifically elucidated and is believed to involve the deactivation of receptor kinases and JAKs. Here we examined the JAK/STAT pathway in MSCs after treated with combined cytokines and found that SHP1 deficiency resulted in robust activation of JAK/STAT pathway. Without SHP1, MSCs could almost cure acute liver injury induced by concanavalin A (ConA) in mice. Thus, we present compelling evidence that SHP1 plays a vital role in regulating the immunosuppressive capacity of mouse MSCs.

## Results

### SHP1 had no obvious effect on the MSC phenotype and proliferation

**Fig. 1:**
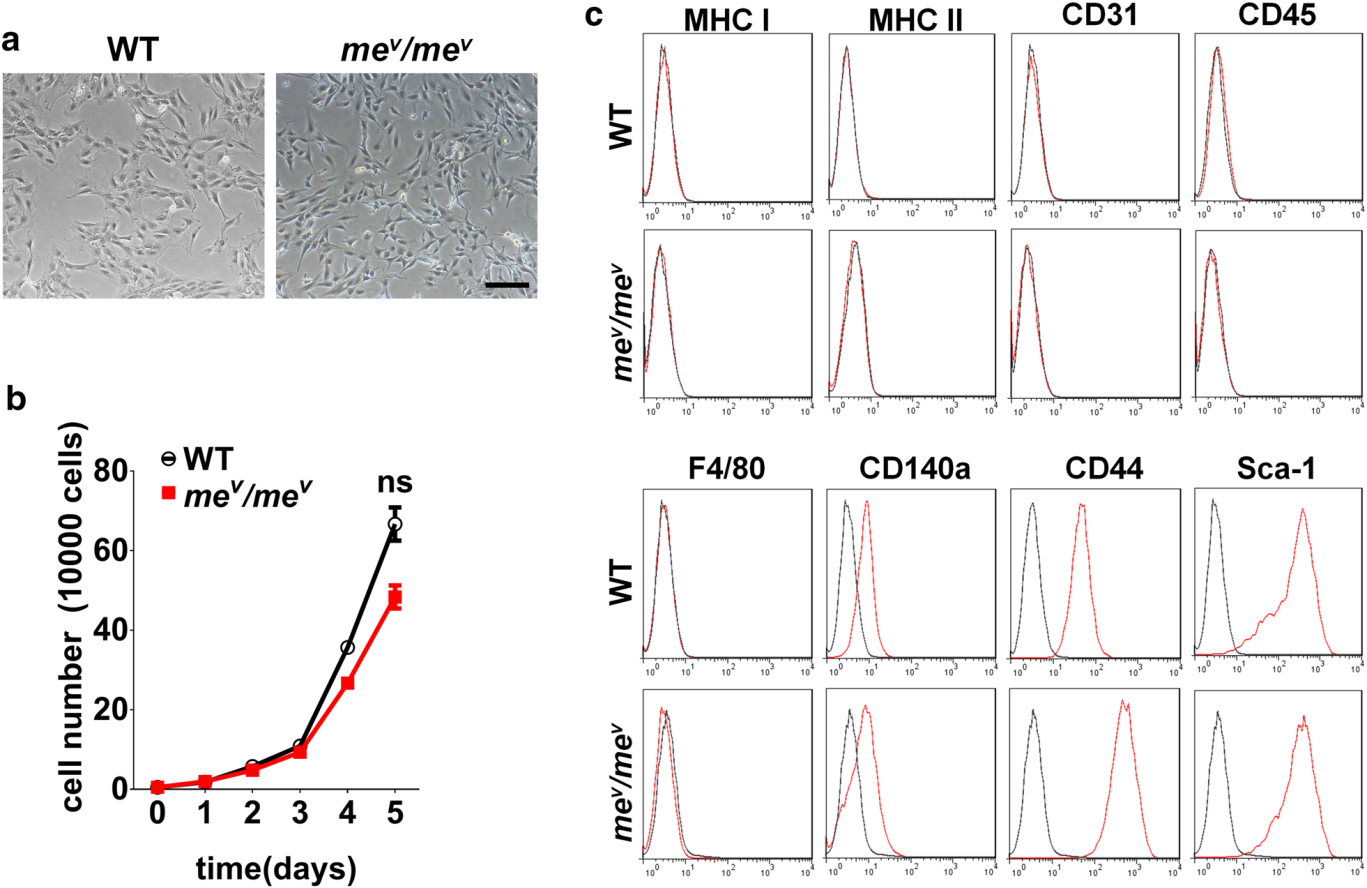
SHP1 deficiency had no effect on the cell morphology, proliferation rate or surface markers. **a** Phase-contrast images of MSCs derived from bone marrow of WT and *me*^*v*^*/me*^*v*^ mice. Scale bar: 50 μm. **b** The same number of WT and *me*^*v*^*/me*^*v*^ MSCs were coated into the 6 well plates, the number were calculated every day until the sixth day and the proliferation rate was monitored. **c** After isolation from bone marrow and culture for an equal number of passages, *me*^*v*^*/me*^*v*^ and WT MSCs were harvested and analyzed for the indicated markers by immunofluorescence staining and flow cytometry. The experiments were repeated at least three times. Error bars represent ± SEM (n = 3), ns, not significant

To study the function of SHP1 in MSCs, bone marrow MSCs were isolated from *me*^*v*^*/me*^*v*^ and WT mice, following our lab established protocol. After culture for several continuous passages, both *me*^*v*^*/me*^*v*^ MSCs and WT MSCs exhibited a similar morphology (Fig. 1a). The proliferation rate of *me*^*v*^*/me*^*v*^ MSCs was also comparable to WT MSCs (Fig. 1b). The cells were also monitored during culture for expression of specific phenotypic markers of MSCs [27, 28]. Immunofluorescence staining and flow cytometric analysis revealed that both *me*^*v*^*/me*^*v*^ MSCs and WT MSCs express CD140a, Stem Cell Antigen 1 (Sca1), CD44 at similar levels, but do not exhibit Major Histocompatibility complex (MHCI, MHCII), CD31, CD45 or F4/80 (Fig. 1c). These results indicate that SHP1 has no effect on the morphology or phenotype of MSCs.

### SHP1 negatively modulates the immunosuppressive capacity of MSCs in vitro

**Fig. 2:**
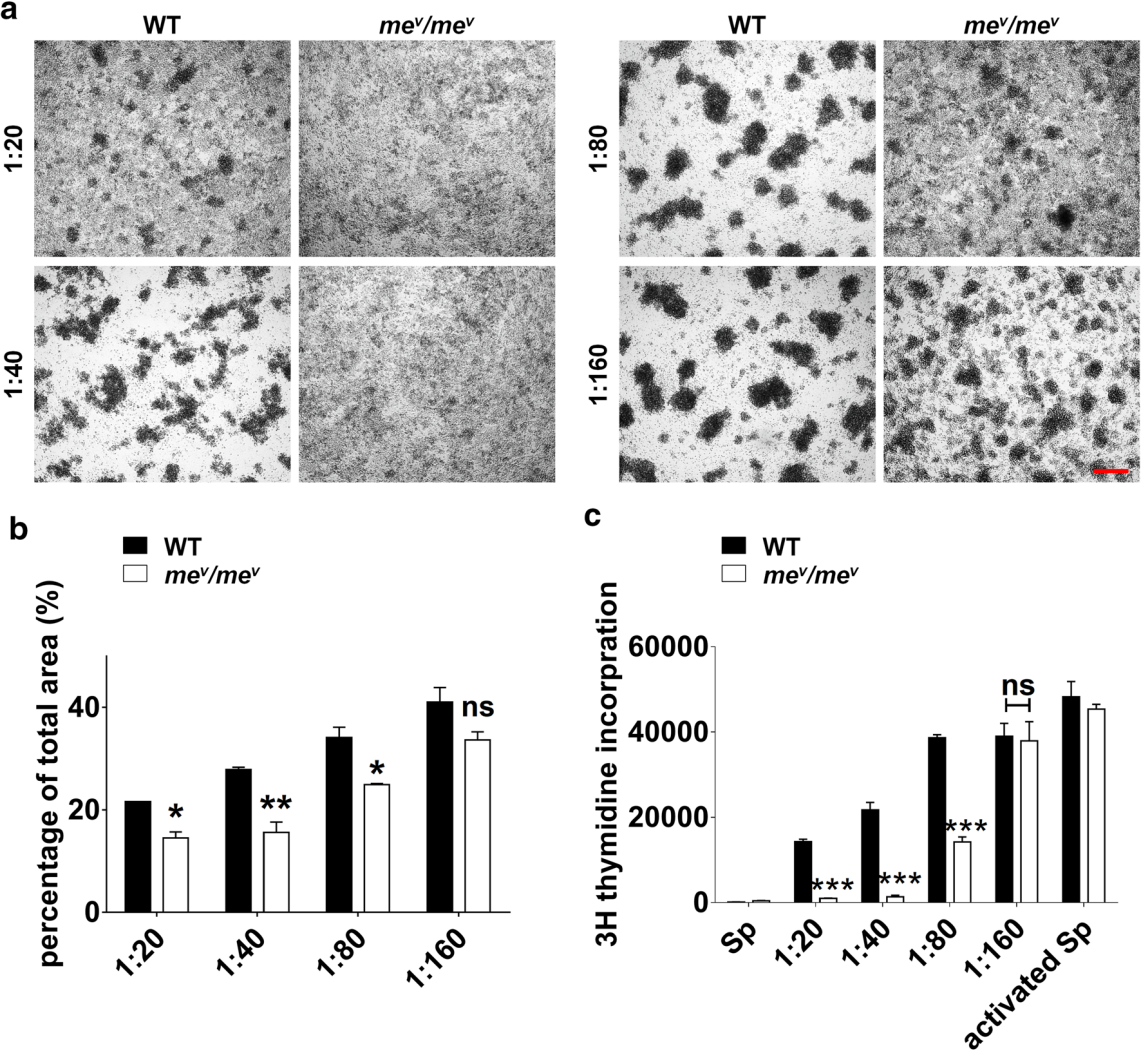
SHP1 deficiency promotes the immunosuppressive effect of MSCs in vitro. Fresh C57BL/6 splenocytes were cocultured with *me*^*v*^*/me*^*v*^ MSCs or WT MSCs and stimulated with anti-CD3 and anti-CD28 (1 μg/ml) each. Various MSC-to-splenocyte ratios were applied as indicated. **a** The extent of cell aggregation was observed by microscope after 48 h. Scale bar: 50 μm. **b** The area of cell clusters was quantitated using ImageJ software. **c** Proliferation was analyzed by ^3^H-Tdr incorporation after 48 h. The experiments were repeated at least three times. Error bars represent ± SEM (n = 3), ns, not significant; *p < 0.05; **p < 0.01; ***p < 0.001

MSCs have been shown to possess potent immunosuppressive capacity in vitro and in vivo [8, 13, 17]. The capacity of MSC immunosuppression is influenced by many factors, such as P53, MiR-155 and so on [29, 30]. Due to SHP1 is a well-known phosphatase mostly blocking downstream signals, we hypothesized that SHP1 could impede the MSC immunosuppression. To test the hypothesis, *me*^*v*^*/me*^*v*^ MSCs and WT MSCs were cocultured with activated splenocytes. The splenocytes were isolated from wild-type mice and stimulated with anti-CD3 and anti-CD28 following a well-established protocol. Different ratios of MSCS to splenocytes were used in this system. The results displayed that at high ratios the splenocyte proliferation were suppressed comparably in both WT and *me*^*v*^*/me*^*v*^ MSCs, whereas at low ratios the splenocyte proliferation was less in *me*^*v*^*/me*^*v*^ MSCs compared to WT MSCs (Fig. 2a). To better quantify the effect, the extent of the areas of cell clusters was calculated using ImageJ software, and the differences were found to be dramatically significant (Fig. 2b). Furthermore, to verify our observation [^3^H] thymidine incorporation was measured to determine splenocyte proliferation. The results exhibited that *me*^*v*^*/me*^*v*^ MSCs were much more efficient in inhibiting the splenocyte proliferation (Fig. 2c). That means SHP1 deficiency enables MSCs possess dramatically enhanced immunosuppressive capacity.

### SHP1 deficiency induced more iNOS and cyclooxygenase 2 (COX2) expression in MSCs

**Fig. 3:**
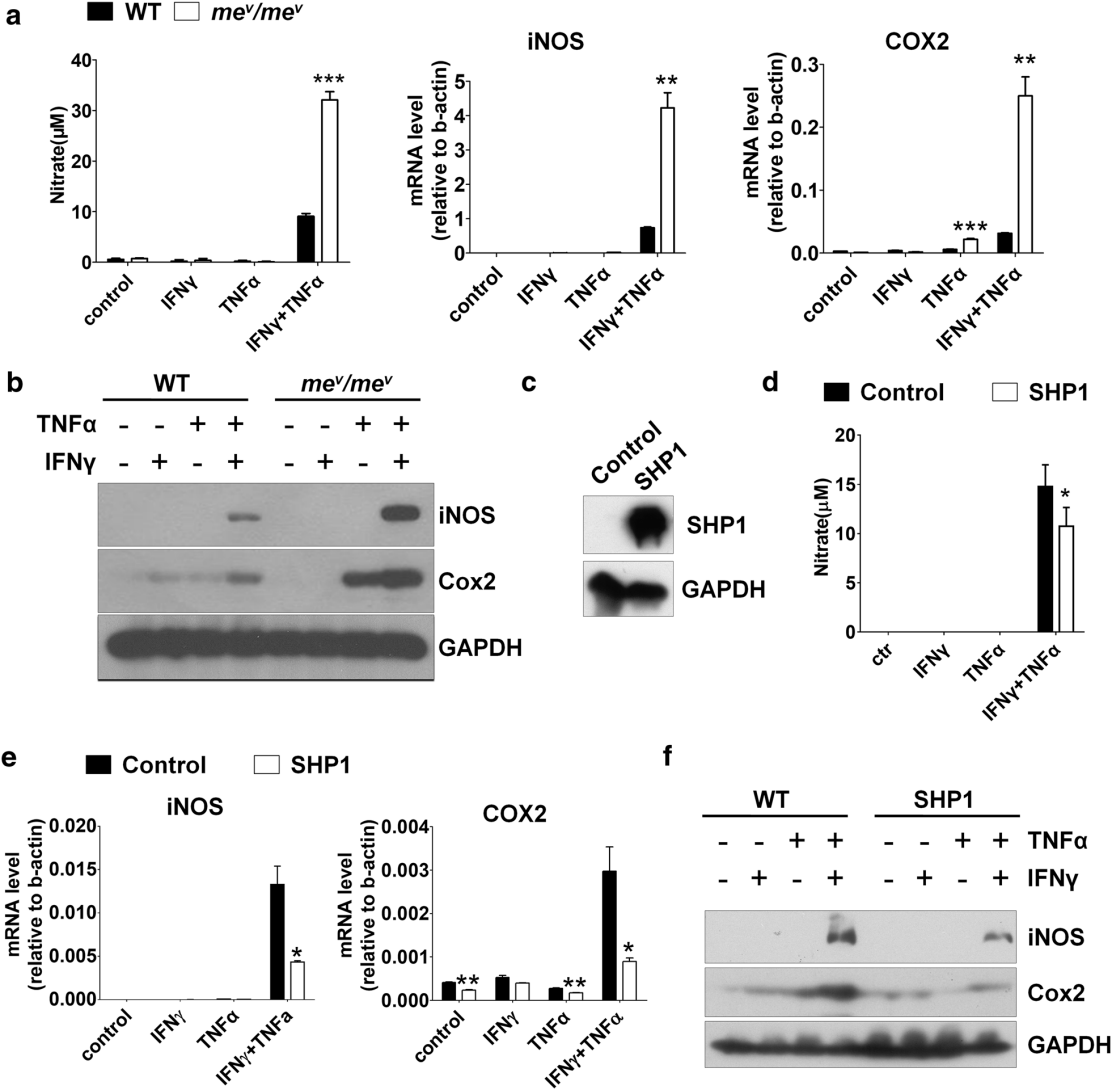
SHP1 regulated NO production under inflammatory cytokines stimulation. *me*^*v*^*/me*^*v*^ MSCs and WT MSCs were treated with IFNγ, TNFα or together (20 ng/ml) for 24 h. **a** The NO content in supernatant was assayed as total nitrate by using modified Griess reagent. The inducible nitric oxide synthase (iNOS) and cyclooxygenase 2 (Cox2) mRNA in *me*^*v*^*/me*^*v*^ MSCs and WT MSCs were analyzed by real-time PCR. **b** The protein level of iNOS and Cox2 in *me*^*v*^*/me*^*v*^ MSCs and WT MSCs were determined by western blotting (WB). **c** WT MSCs were transfected by lentivirus containing an SHP1-expression vector or control vector, and SHP1 expression was measured by WB. **d** The NO content in supernatant was assayed as total nitrate by using modified Griess reagent. **e** The mRNA level of iNOS and Cox2 were analyzed by real-time PCR. **f** The protein level of iNOS and Cox2 were analyzed by WB. The experiments were repeated at least three times. Error bars represent ± SEM (n = 3), ns, not significant; *p < 0.05; **p < 0.01; ***p < 0.001

Our previous studies have shown that the immunosuppressive function of murine MSCs was achieved by producing large amounts of NO after primed by inflammatory cytokines [12]. Therefore, to examine whether the effect of SHP1 on immunosuppression of MSCs is exerted through regulating NO production, *me*^*v*^*/me*^*v*^ MSCs and WT MSCs were treated with IFNγ and TNFα for 24 h, and the supernatant nitrate concentration of stimulated MSCs was determined by Griess reagent. Indeed, *me*^*v*^*/me*^*v*^ MSCs produced markedly more NO compared to WT MSCs (Fig. 3a). The NO production is specifically influenced by iNOS expression in MSCs, since MSCs could not express neuronal NOS and endothelial NOS. Therefore, the mRNA and protein level of iNOS was examined by real-time PCR and western blotting respectively. Consistently, the mRNA and protein level of iNOS dramatically increased in *me*^*v*^*/me*^*v*^ MSCs compared to WT MSCs (Fig. 3a, b). As previous reported, PGE2 has been shown to play a vital role in the immunomodulation of MSCs [31, 32]. In addition, PGE2 is synthesized by COX2 which is produced by MSCs under inflammatory cytokine stimulus [14, 33]. Therefore, in the current study the expression of COX2 was also determined by real-time PCR and western blotting respectively. Similarly, compared to WT MSCs the mRNA and protein level of COX2 also remarkably increased in *me*^*v*^*/me*^*v*^ MSCs (Fig. 3a, b). In hence, SHP1 inhibits the immunosuppressive capacity of MSCs by reducing iNOS and COX2 expression.

### Overexpression of SHP1 suppressed iNOS and COX2 production in MSCs

To further verify the function of SHP1 in MSCs, WT MSCs were transfected with a constitutive SHP1 expression cassette, and the expression of SHP1 was examined by western blotting (Fig. 3c). When the SHP1-overexpressing MSCs were stimulated with IFNγ and TNFα, we observed NO production in the supernatant was reduced compared to WT control (Fig. 3d). Furthermore, the mRNA and protein level of iNOS and COX2 was examined by real-time PCR and western blotting respectively. In consistent, both the mRNA and protein level of iNOS and COX2 decreased significantly compared to WT control (Fig. 3e, f). These results further illustrate the important role of SHP1 in regulating immunosuppression of MSCs.

### SHP1 regulates MSC immunosuppression by JAK-STAT and p38 signals

**Fig. 4:**
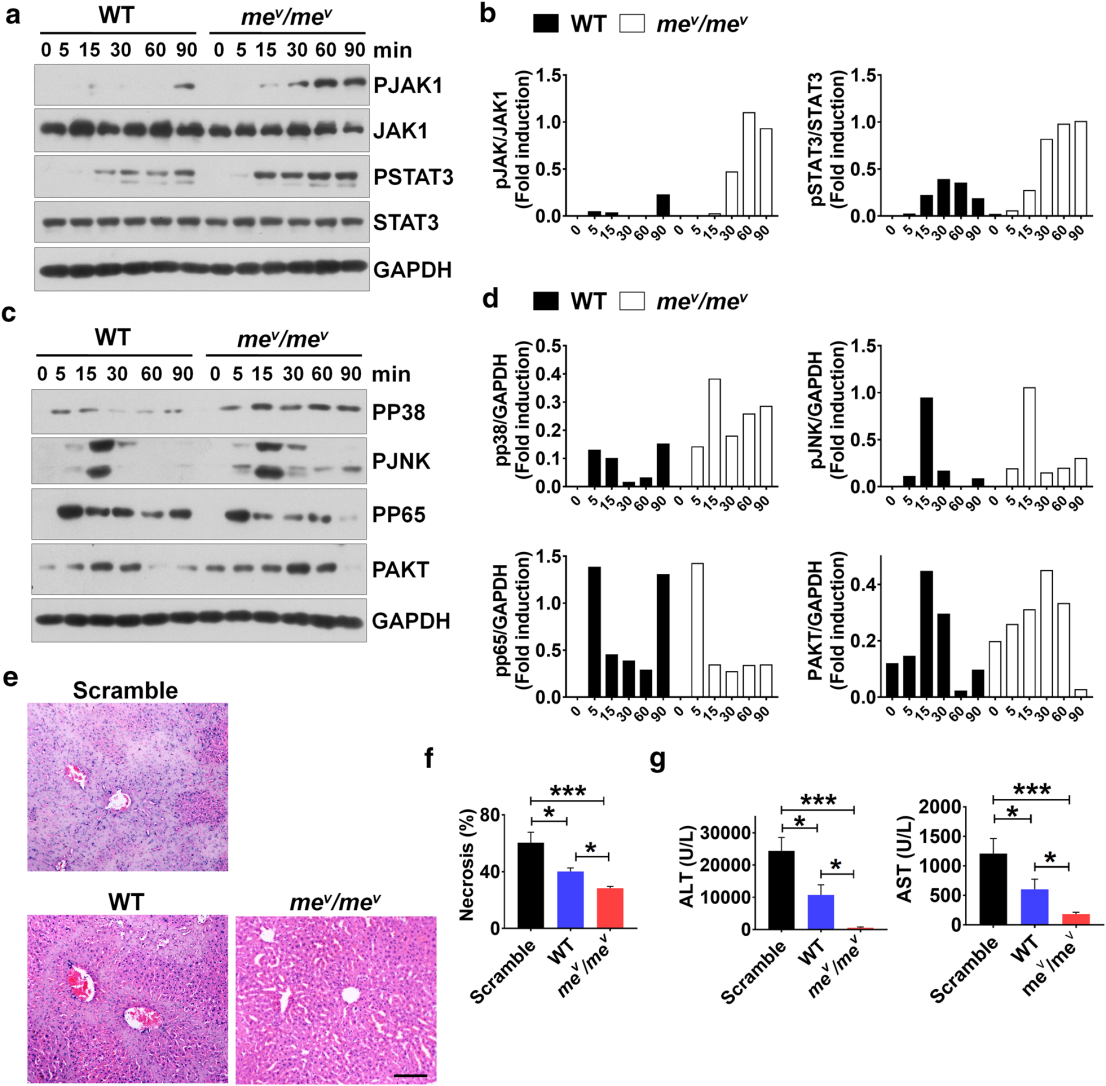
SHP1 promotes NO production through JAK1/STAT3 signals, and *me*^*v*^*/me*^*v*^ MSCs alleviated ConA-induced liver injury more obviously compared to WT MSCs. WT and *me*^*v*^*/me*^*v*^ MSCs were treated with IFNγ and TNFα (20 ng/ml) for the indicated times. **a** The phosphorylated JAK1 and STAT3 as well as total JAK1 and STAT3 were evaluated by WB. **b** The gray intensity of pJAK1 and pSTAT3 was calculated by ImageJ software. **c** The phosphorylated P38, JNK, P65 and AKT were determined by WB. **d** The gray intensity of PP38, PP65 and PAKT was calculated by ImageJ software. ConA (15 mg/kg) was intravenously injected with WT or *me*^*v*^*/me*^*v*^ MSCs, and the serum and liver samples were isolated 8 h later. **e** The liver sections were stained by hematoxylin & eosin. Scale bar: 50 μm. **f** The area of necrosis in liver was calculated by ImageJ software. **g** The levels of ALT and AST in serum were measured. The experiments were repeated at least three times. Error bars represent ± SEM (n = 5), ns, not significant; *p < 0.05; **p < 0.01; ***p < 0.001

Previous reports have shown that SHP1 negatively regulates various signaling pathways, including JAK/STAT signals [34, 35]. Therefore, to define the mechanism of SHP1 regulating iNOS and COX2 expression in MSCs, *me*^*v*^*/me*^*v*^ MSCs and WT MSCs were treated with IFNγ and TNFα for indicated time, and the signal molecules were measured by western blotting. We observed that both JAK1 phosphorylation at tyrosine 1022/1023 (Y1022/1023) and STAT3 phosphorylation at tyrosine 705 (Y705) were dramatically increased in *me*^*v*^*/me*^*v*^ MSCs compared to WT MSCs, although total level of JAK1 and STAT3 were no different (Fig. 4a). To better quantify the gray intensity, the blots were calculated using ImageJ software. We found that the levels of JAK1 and STAT3 phosphorylation were highly increased (Fig. 4b, d).

Considering that various signals are involved in regulating expression of iNOS, therefore, the phosphorylation of p38, JNK, NF-κB and AKT was also examined by western blotting. And the results displayed that the phosphorylation of p38 dramatically increased in *me*^*v*^*/me*^*v*^ MSCs compared to WT MSCs, although the phosphorylation of JNK, NF-κB and AKT no obvious difference (Fig. 4c). To better quantify the gray intensity, the blots were calculated using ImageJ software. We found that the levels of P38 phosphorylation were highly increased, and the levels of p65 and Akt phosphorylation were comparative between WT and mev/mev MSCs (Fig. 4d). These results indicate that SHP1 modulated the expression of iNOS and COX2 mainly through regulating JAK-STAT and p38 signals.

### *me*^*v*^*/me*^*v*^ MSCs alleviated ConA-induced liver injury more effectively than WT MSCs

ConA was illustrated to stimulate T cell activation, which was established as the major effector cells in liver injury [36], resulting in acute immune response in liver [37]. The intravenous injection of ConA was widely used as model for acute immune-mediated hepatitis [38]. Therefore, ConA-induce liver injury was an ideal model to investigate the immunosuppressive effect of MSCs in vivo. To investigate the role of SHP1 in regulating immunosuppression of MSCs in vivo, the model of ConA-induced liver injury was employed in our studies, while the WT or *me*^*v*^*/me*^*v*^ MSCs were also transferred into mice via tail vein. Interestingly, WT MSCs slightly alleviated liver injury, while *me*^*v*^*/me*^*v*^ MSCs almost eliminated the liver damage according the H&E staining images (Fig. 4e). To better quantify the effect, the extent of the necrotic areas was calculated using ImageJ software, and the differences were found to be highly significant (Fig. 4f).

The liver could produce some enzymes, such as Alanine Aminotransferase (ALT) and Aspartate Aminotransferase (AST), which were signs of liver problem and could be measured via blood test. To confirm the recovery of liver damage by MSCs, the levels of ALT and AST in serum were determined. Consistently, the administration of *me*^*v*^*/me*^*v*^ MSCs tremendously reduced the serum level of ALT and AST compared to the groups of PBS or WT MSCs treatment (Fig. 4g). The results indicated that SHP1 was critical in modulating the immunosuppression of MSCs in vivo.

## Discussion

MSCs are multipotent stromal cells and can be isolated from various tissues. Owing to the self-renewal capacity and pluripotent differentiation, MSCs exhibit great potential for application in degenerative diseases and immune disorders. Although the physiological characters and function of MSCs in vivo are still elusive, the in vitro expanded MSCs have achieved therapeutic effects through immunosuppressive capacity and regenerative potentials. Numerous reports have suggested that MSCs affect the adaptive and innate immune systems through regulating the immune T cell and B cell response, inhibiting maturation, activation and antigen presentation of dendritic cells and blocking NK cell activation [39–43]. However, the mechanism regulating the MSC immunomodulation is still elusive [44, 45]. In our study, we found that SHP1 deficient MSCs acquired robust immunosuppressive capacity compared with WT MSCs. The effects of SHP1 on MSC immunosuppression are exerted through regulating the expression of iNOS, and are linked to inhibition of JAK/STAT and p38 pathway.

The immunosuppressive capacity of MSCs is shown to be non-intrinsic but is licensed after activation with inflammatory cytokines. Pretreated with IFNγ combined with TNFα, IL1α or IL1β, MSCs are elicited high levels of immunosuppressive factors, as well as a burst of chemokines and adhesion molecules [12]. Therefore, the effect of SHP1 deficiency on the immunosuppressive capacity of MSCs was examined in vitro, using cells form SHP1-deficient mice. In this study, we found that SHP1 deficiency resulted in dramatically increase of MSC immunosuppression. The immunosuppression induced by MSCs was mediated through different molecules in different species [15]. NO produced by iNOS is a major player in the process of murine MSC immunosuppression [12]. A decrease of NO level in serum was also observed after MSC administration [46]. When expression of iNOS after treatment with inflammatory cytokines was analyzed by WB and real-time PCR, iNOS was found to be notably increased in SHP1-deficient MSCs at both the protein and mRNA levels. PGE2 is the major prostaglandin generated by COX2 and exerts a vital role in the immunosuppression of MSCs. After treatment with inflammatory cytokines, the protein and mRNA levels of COX2 were also significantly increased in SHP1-deficient MSCs.

Various pathways are implicated in transmitting extracellular signals to the nuclei for iNOS gene expression [47], including NF-κB, JAK/STAT and MAPK signaling [48–50]. Treatment of intestinal epithelial cells with IFNγ and TNFα induced the expression of iNOS through JAK/STAT1 signaling [51]. STAT family members, such as STAT1 and STAT3, have been shown to induce the expression of iNOS and COX2 in pheochromocytoma cell line PC12 [50]. In the present study, when treated with IFNγ combined with TNFα, *me*^*v*^*/me*^*v*^ MSCs displayed remarkable more phosphorylation of JAK1 at Tyr1022/1023 and STAT3 at Tyr705. Previous studies have shown a prominent role for p38 MAPK-mediated signaling in the control of iNOS expression using a specific inhibitor of the kinase, SB203580 [52, 53]. Treatment with IFNγ and TNFα resulted in iNOS expression in glioma cells, which was mediated by p38 MAPK [49]. Therefore, to verify the role of SHP1, we analyzed the p38 signaling in SHP1-deficient MSCs derived from *me*^*v*^*/me*^*v*^ mice. Consistent with previous reports, P38 signaling was found to be down regulated by SHP1.

ConA-induced acute hepatitis, which closely mimics the pathogenesis and pathological characteristics of autoimmune hepatitis patients [36], has been regarded as a typical and well-established model for liver injury predominantly mediated by acute inflammation. ConA-induced liver injury is predominantly driven by the activation of T cells recruited into the liver [54]. MSCs has been explained to alleviate ConA-induced acute liver injury [55]. In the present study, the role of SHP1 in modulating MSC-based therapy of liver injury was investigated. We found that WT MSCs lightly attenuated the liver damage induced by ConA, whereas SHP1 deficiency boosted the therapeutic effect of MSCs on liver injury. The results indicated that SHP1 played a pivotal role of immunoregulation of MSCs and highly contributed to the protective effect of MSCs in the animal model.

## Conclusions

This study suggests SHP1 regulates the immunosuppressive capacity of MSCs in vitro *and* in vivo, and this effect is exerted through inhibiting iNOS expression modulated by JAK1/STAT3 and P38 signals.

## Methods

### Mice and cells

*me*^*v*^*/me*^*v*^ mice (C57BL/6J background, Stock No: 000811) were purchased from the Jackson Laboratory. According to our previous established protocol [9], the tibias and femurs of 6- to 10-week-old WT and age-matched *me*^*v*^*/me*^*v*^ mice were dissected from the surrounding tissues, and the MSCs were generated from the bone marrow cells. The cells were cultured in low glucose DMEM supplemented with 10% heat-inactivated FBS, 2 mM glutamine, 100 U/ml penicillin, and 100 μg/ml streptomycin (all from Invitrogen, Carlsbad, CA, USA). After 24 h, non-adherent cells were discarded and adherent cells were maintained with medium replenishment every 3 days. The splenocytes were maintained in RPMI-1640 medium supplemented with 10% fetal bovine serum, 2 mM glutamine, 100 U/ml penicillin and 100 mg/ml streptomycin, and were activated with soluble anti-CD3 and anti-CD28 (1 μg/ml, each) for 2 days.

### Reagents

FBS, DMEM low-glucose medium, DMEM high-glucose medium, RPMI-1640medium, trypsin–EDTA, glutamax and penicillin–streptomycin solution were purchased from Invitrogen (Carlsbad, CA, USA). Recombinant mouse IFNγ and TNFα were purchased from R&D Systems (Minneapolis, MN); Griess reagent were obtained from Sigma-Aldrich (St. Louis, MO). Antibodies used in flow cytometry, including PE-conjugated anti-mouse CD31 (Product code: 11031182), CD34 (Product code: 11034182), Sca-1 (Product code: 12598182), CD44 (Product code: 12044182), CD45 (Product code: 12045182), MHC I KbDb (Product code: 12599882), F4/80 (Product code: 12480182), FITC-conjugated anti-mouse MHC class II I-A (Product code: 12532281), and APC-conjugated platelet-derived growth factor receptor α (PDGFRα) (Product code: 17140181) were purchased from eBioscience (San Diego, CA, USA). Antibodies used in western blotting analysis were: SHP1 (Product code: ab32559), and COX2 (Product code: ab15191) (Abcam, Cambridge, MA, USA), iNOS (Product code: 39898), JAK1 (Product code: 50996), pJAK1 (Product code: 3331), STAT3 (Product code:, pSTAT3 (Product code: 9139), pP38 (Product code: 9216), pJNK (Product code: 9255), pP65 (Product code: 3039), pAKT (Product code: 4060) and GAPDH (Product code: 5174) (Cell Signaling Technology, Danvers, MA, USA).

### Flow cytometry

After mixed with specific antibodies, the cell suspension was incubated for 30 min on ice. After washed three times by PBS, the samples were analyzed by FACS Calibur flow cytometer (BD Biosciences, San Jose, CA, USA). FlowJo 7.6.1 software was used for data analysis.

### Proliferation assay

The spleen was isolated from 6 to 8 weeks old mice, and the splenocytes were collected via 70 μm cell strainer. The splenocytes were co-cultured with MSCs for 48 h in 96 well plates at different ratios. For proliferation assay, 1μ Ci ^3^H-thymidine was (Tdr, Shanghai Institute of Applied physics, Chinese Academy of Sciences, China) added to each well for 4 to 6 h before the cell cultures were frozen. The radioactivity of ^3^H-thymidine was measured by scintillation counter (Perkin-Elmer, Waltham, MA, USA).

### Nitrate detection by Griess test

The modified Griess reagent was applied to determine the NO production. Culture supernatant and standards (50 μl) were added into plat-bottom 96-well plate, and then the Griess reagent (50 μl) was added. After incubated for 5 min at room temperature, the amount of nitrate was measured colorimetrically by absorbance at 540 nm.

### SHP1 overexpression

The mouse SHP1 mRNA sequence was amplified from cDNA by PCR using PrimerSTAR (TAKARA, Dalian, China), and the PCR product was inserted into the PLVX-IRES-PURO plasmid after double digestion. The constructed plasmid combined with PMD2G and PSPAX2 plasmids was transfected to 293T cells by LIPO2000 (Invitrogen, Carlsbad, CA, USA), and the supernatant was collected after 48 h. The supernatant was filtered through a 0.22 μm filter to eliminate cell debris, and then was concentrated by centrifugation. The lentivirus with polybrene (5 μg/ml) was applied to transfect WT MSCs for 12 h, and the transfected MSCs were purified using puromycin (2 μg/ml). The expression of SHP1 protein in purified MSCs was detected by western blotting.

### Western blotting

MSCs were cracked by RIPA lysis buffer (Biyotime) for 30 min on ice, and protein concentration was measured with the BCA kit (Bio-Rad). The 4× loading buffer was added into protein samples, and after boiled for 10 min at 100 °C the samples were separated by SDS-PAGE, transferred to a nitrocellulose membrane, and blocked with 5% skim milk. The blots were incubated with specific primary antibodies overnight followed with anti-rabbit-HPR, and staining detected with the ECL system (Millipore Corporation, Billerica, USA).

### Real-time PCR

Total RNA was extracted using TRIzol (Invitrogen). First-strand cDNA was reverse-transcribed using cDNA Synthesizing Kit (Takara, Dalian, China) according to the manufacturer’s instructions. The expression profiles of specific genes were determined with SYBR Green reagent (Roche). Gene expression was normalized to endogenous β-actin mRNA.

### Animal model of liver injury induced by ConA

The mice were intravenously injected with ConA (15 mg/kg) to cause acute liver injury, meanwhile PBS, WT MSCs or *me*^*v*^*/me*^*v*^ MSCs were also intravenously injected, according to the previous protocol established by our lab. The serum and liver tissue were collected after 8 h. Then the serum levels of ALT and AST were determined by the detection kits. The liver tissues were fixed with PFA overnight, embedded in paraffin, sectioned and stained with hematoxylin & eosin to observe the pathological changes.

### Statistical analysis

Statistical analysis was performed using Prism 5.0 software (GraphPad Software, Inc.). Unpaired two-tailed Student’s t test was used in all instances, and statistical significance is reported as follows: ns, not significant; *p < 0.05; **p < 0.01; **p < 0.001.

## Data Availability

All data generated or analyzed during this study are included in this published article (and its additional files).

## Abbreviations

MSCs: Mesenchymal stromal cells
SHP1: SH2 domain-containing phosphatase-1
COX2: Cyclooxygenase 2
NO: Nitric oxide
PGE2: Prostaglandin E2
IDO: Indoleamine 2,3-dioxygenase
TGFβ1: Transforming growth factor-β1
TSG6: Tumor Necrosis factor-a (TNF-a)-stimulated gene 6 protein
PD-L1: Programmed death-ligand
ConA: Concanavalin A
Sca1: Stem cell antigen 1
ALT: Alanine aminotransferase
AST: Aspartate aminotransferase
